# Development of a parental report questionnaire for restless legs syndrome (RLS) in children: the *RLSQ*

**DOI:** 10.1186/1757-1146-4-S1-O15

**Published:** 2011-05-20

**Authors:** Angela Evans, Sarah Blunden

**Affiliations:** 1 School of Health Science, University of South Australia, Adelaide, SA, 5000, Australia; 2 Centre for Sleep Research, University of South Australia, Adelaide, SA, 5000, Australia

## Background

Restless legs syndrome (RLS) in children is commonly reported, yet frequently undiagnosed. RLS can cause significant sleep disturbance and its associated deficits may have cardiovascular and neurocognitive consequences. Growing pains (GP) is often confused or synonymous with RLS, yet has been better researched and can be identified by parental questionnaire. RLS has not been able to be so distinguished, which renders an outstanding need. Therefore this study aimed to develop and validate a questionnaire to identify RLS, in children. The significance of this project is that RLS in children will be better identified in children for the first time.

## Methods

A process of triangulation was undertaken to develop the RLS questionnaire. The literature, parent interviews and a children’s focus group were the sources of initial data. Themes were extracted by independent review of the transcripts and the questionnaire was subsequently constructed and validated. The reliability of the questionnaire was examined using a same subject, repeated measures study.

## Results

The interviews covered the parent’s accounts of RLS in six children (two girls, four boys) all aged between eight and 10 years. The focus group obtained the experience of children suffering RLS. A questionnaire of 11 questions was developed and validated from a small convenience sample (*n*= 11). Internal consistency yielded 65% and repeat measures reliability *rho* = 0.58.

## Conclusions

The questionnaire developed enables RLS to be identified in children specifically and for the first time. Such instrumentation may be used to establish prevalence, discriminate RLS from GP, to evaluate management programs and to assist treating clinicians.

